# Efficacy of trivalent influenza vaccine against laboratory-confirmed influenza among young children in a randomized trial in Bangladesh

**DOI:** 10.1016/j.vaccine.2017.10.074

**Published:** 2017-12-15

**Authors:** Melissa A. Rolfes, Doli Goswami, Amina Tahia Sharmeen, Sultana Yeasmin, Nasrin Parvin, Kamrun Nahar, Mustafizur Rahman, Marion Barends, Dilruba Ahmed, Mohammed Ziaur Rahman, Joseph Bresee, Stephen Luby, Lawrence H. Moulton, Mathuram Santosham, Alicia M. Fry, W. Abdullah Brooks

**Affiliations:** a Epidemic Intelligence Service, Centers for Disease Control and Prevention, Atlanta, GA, USA; b Influenza Division, Centers for Disease Control and Prevention, Atlanta, GA, USA; c Icddr,b, Dhaka, Bangladesh; d Stanford Medical School, Stanford, CA, USA; e Johns Hopkins Bloomberg School of Public Health, Baltimore, MD, USA

**Keywords:** Influenza, Vaccine, Clinical trial, Children

## Abstract

**Background:**

Few trials have evaluated influenza vaccine efficacy (VE) in young children, a group particularly vulnerable to influenza complications. We aimed to estimate VE against influenza in children aged <2 years in Bangladesh; a subtropical setting, where influenza circulation can be irregular.

**Methods:**

Children aged 6–23 months were enrolled 1:1 in a parallel, double-blind, randomized controlled trial of trivalent inactivated influenza vaccine (IIV3) versus inactivated polio vaccine (IPV); conducted August 2010–March 2014 in Dhaka, Bangladesh. Children received two pediatric doses of vaccine, one month apart, and were followed for one year for febrile and respiratory illness. Field assistants conducted weekly home-based, active surveillance and ill children were referred to the study clinic for clinical evaluation and nasopharyngeal wash specimen collection. Analysis included all children who received a first vaccine dose and compared yearly incidence of reverse transcription polymerase chain reaction (RT-PCR)-confirmed influenza between trial arms. The VE was estimated as 1 − (rate ratio of illness) × 100%, using unadjusted Poisson regression. The trial was registered with ClinicalTrials.gov, number NCT01319955.

**Results:**

Across four vaccination rounds, 4081 children were enrolled and randomized, contributing 2576 child-years of observation to the IIV3 arm and 2593 child-years to the IPV arm. Influenza incidence was 10 episodes/100 child-years in the IIV3 arm and 15 episodes/100 child-years in the IPV arm. Overall, the VE was 31% (95% confidence interval 18, 42%) against any RT-PCR-confirmed influenza. The VE varied by season, but was similar by influenza type/subtype and participant age and sex.

**Conclusions:**

Vaccination of young children with IIV3 provided a significant reduction in laboratory-confirmed influenza; however, exploration of additional influenza vaccine strategies, such as adjuvanted vaccines or standard adult vaccine doses, is warranted to find more effective influenza vaccines for young children in low-income countries.

## Highlights

• There is limited data on efficacy of yearly influenza vaccination in children aged <2 years.
• Influenza vaccination was found to be safe and significantly reduced influenza in young children.
• These findings support yearly influenza vaccination of young children.

## 1 Introduction

Children under 2 years old are at increased risk for serious influenza complications, including pneumonia, resulting in increased rates of hospitalization and mortality [1], [2]. For this reason, the Strategic Advisory Group of Experts (SAGE) on Immunization of the World Health Organization (WHO) have identified children under 2 years as a priority group to receive seasonal influenza vaccination [3].

The trivalent inactivated influenza vaccine (IIV3) is pre-qualified by the World Health Organization or licensed in the United States for children under 2 years old and is indicated for children as young as 6 months old. However, in three randomized trials, the efficacy of IIV3 against laboratory-confirmed influenza has been inconsistent in this age group [4], [5], [6]. Only one trial has included children in resource-constrained settings [6]. No trials of IIV3 have been published from tropical or subtropical settings where influenza circulation tends to be seasonal but can be irregular or occur year-round. We aimed to evaluate the efficacy of IIV3 in children 6–23 months old living in Dhaka, Bangladesh through a parallel double-blind randomized controlled trial. There were co-primary outcomes of the trial; clinical pneumonia and laboratory-confirmed influenza. The clinical pneumonia outcome will be discussed elsewhere and the remainder of this manuscript is focused on laboratory-confirmed influenza and the safety profile of IIV3.

## 2 Methods

### 2.1 Study design and participants

We recruited healthy children, aged 6–23 months, from the Kamalapur community, an icddr,b surveillance area of Dhaka, Bangladesh that has been engaged in active respiratory disease surveillance activities since 1999 [7], [8], [9], to participate in a multi-year, parallel, double-blind randomized controlled vaccination trial (NCT01319955, www.clinicaltrials.gov). Approval to conduct the trial was granted by the institutional review boards at icddr,b in Dhaka and Johns Hopkins University in Baltimore, MD. The Centers for Disease Control and Prevention, in Atlanta, GA, approved a request to rely on the approval at icddr,b.

To recruit children into the trial, field research assistants visited households enrolled in the ongoing surveillance activities [7], [8], [9] and described the trial to parents. Children, aged 6–23 months, were brought to the study clinic in Kamalapur for eligibility screening. Children were excluded if they had chronic disease (cardiac, respiratory, or neurologic), had a family history of confirmed or suspected tuberculosis, had severe malnutrition requiring hospitalization, had an egg allergy, required hospitalization for any reason, or were currently enrolled in another clinical trial. Clinic staff sought informed consent from parents/guardians for eligible children after describing the nature and consequences of the trial, and, then afterwards, enrolled, randomized, and vaccinated consented children at the same visit. Children with a current acute illness were asked to return seven days later for re-evaluation and vaccination. Only one child per household was enrolled. Children were eligible to enroll in more than one season if they continued to meet eligibility criteria at the time of vaccination for subsequent seasons.

### 2.2 Randomization and masking

Children were randomized 1:1 to receive trivalent inactivated influenza vaccine (IIV3) or inactivated polio vaccine (IPV) using permuted random blocks of size two to eight. Vaccine allocations were assigned to sequential numbers using random number tables. The sequential numbers were printed on opaque labels that were placed on pre-filled vaccine syringes by the study data management team who were not directly involved in vaccination and post-vaccine evaluation process. Labels concealed the volume of vaccine in each syringe. The vaccinators were not involved in any level of participant follow-up or clinical care. Randomization occurred during a child’s first season of enrollment and vaccine assignment remained the same for all subsequent seasons of enrollment.

### 2.3 Vaccine

At the time of the trial, trivalent inactivated influenza vaccine was licensed for use in children aged ≥6 months in Bangladesh but was not included in the list of recommended vaccines; and inactivated polio vaccine was not included in the routine childhood immunization schedule. Vaccines were donated by Sanofi Pasteur. The composition of the influenza vaccine followed recommendations from the World Health Organization for the southern hemisphere. During the 2010, 2011, and 2012 seasons, IIV3 included an A/California/7/2009 (H1N1)-like virus, an A/Perth/16/2009 (H3N2)-like virus, and a B/Brisbane/60/2008-like virus (Victoria lineage). In 2013, IIV3 components were changed to include an A/California/7/2009 (H1N1)-like virus, an A/Victoria/361/2011 (H3N2)-like virus, and a B/Wisconsin/1/2010-like virus (Yamagata lineage). Inactivated polio vaccine (IPV) was the active control vaccine. Children received 0.25 mL of IIV3 and 0.50 mL of IPV into the thigh muscle using a 25 gauge, 16 mm needle (vaccine lot numbers are provided in Supplemental Table 1). During a first season of enrollment, children received two doses of vaccine, one month apart. During subsequent seasons of enrollment, children received a single dose of vaccine. Children did not receive any other vaccine during the visit in which they were vaccinated with IIV3 or IPV.

### 2.4 Influenza seasons

In Bangladesh, influenza is seasonal with peak activity occurring between May and September each year [10], [11]. For the purposes of the analysis, year-long seasons were defined from April through the following March. Enrollment and vaccination occurred over four influenza seasons with the goal to complete vaccination by March 31 each year. Delays in vaccine delivery occurred in 2010, consequently vaccination for the first season began on August 30 and 95% of doses were delivered by October 4, 2010. In subsequent years, 95% of vaccinations with a first dose were completed by March 22, 2011 (95% of second doses were completed by May 5, 2011) for the second season, March 28, 2012 (95% of second doses were completed by May 13, 2012) for the third season, and by April 28, 2013 (95% of second doses were completed by June 6, 2013) for the fourth season.

### 2.5 Surveillance for influenza

Active surveillance for respiratory and febrile illness was conducted from August 30, 2010 through March 31, 2014. Field research assistants made weekly visits to children’s homes to measure axillary temperature and respiratory rate and inquire about signs and symptoms of illness in the enrolled child during the prior week. Children who had experienced an illness or exhibited one major or two minor signs of illness during the visit were referred to the study clinic for further evaluation. Major signs included fever, age-specific tachypnea using WHO criteria (≥50 breaths/min for children 6–11 months and ≥40 breaths/min for children 12–23 months), danger signs (chest in-drawing, lethargy, cyanosis, inability to drink, or convulsions), difficult breathing, noisy breathing, and ear pain/discharge. Minor signs included cough, rhinorrhea, sore throat, myalgia/arthralgia, chills, headache, irritability/decreased activity, and vomiting. Parents were also encouraged to bring the child to a study clinic whenever illness occurred.

Trained physicians conducted clinical exams on ill children who presented to care or were referred to care by field research assistants. Nasopharyngeal wash specimens were collected as previously described [9], [12]. Briefly, specimens were collected from all participants with a documented fever (axillary temperature ≥38 °C) or who met pre-specified clinical case definitions, including pneumonia, upper respiratory infection, otitis media, sinusitis, fever without localizing signs, or bronchiolitis (Supplemental Table 2). Field research assistants visited children with a recent illness at home daily until the resolution of the illness and until children were seen for a convalescent exam at the study clinic, which occurred at the end of each illness episode.

Nasopharyngeal wash specimens were sent to the Virology Laboratory at icddr,b in Dhaka for influenza detection using real-time reverse transcription polymerase chain reaction (RT-PCR) [13]. The RT-PCR primers, probes and testing protocol were developed and provided by the CDC Influenza Division, and designed for universal detection of influenza A and B viruses and influenza A virus subtypes (A(H1N1)pdm09 and A(H3N2)). A house-keeping gene, RNP, was used to check sample quality, check proper nucleic acid extraction, and to monitor PCR inhibition. A negative control and a CDC provided positive control were included in every RT-PCR run.

Viral isolates, from cell cultures on Madin Darby canine kidney cell lines, were antigenically characterized using hemagglutinin inhibition (HAI) assays [14]. Viral isolates were considered similar to the vaccine (i.e. vaccine-like) if antisera raised against the vaccine virus antigen reacted with the isolate with an HAI titer that was at least 4-fold higher than the reaction to other reference antisera [14]. Reference antisera were provided by the World Health Organization as part of international influenza surveillance efforts.

Routine community surveillance for influenza was conducted in the Kamalapur area throughout the trial period, as previously reported [9], and included RT-PCR detection of influenza as well as antigenic characterization, as described above.

### 2.6 Clinical outcome

The primary outcome of the influenza component of the trial was laboratory-confirmed influenza. Influenza, including influenza subtypes A(H1N1)pdm09, A(H3N2), and B, was detected in nasopharyngeal wash specimens, using RT-PCR assays conducted in the Virology Laboratory at icddr,b in Dhaka. Clinical pneumonia, with or without wheeze, was defined as age-specific tachypnea (≥50 breaths/min for children 6–11 months and ≥40 breaths/min for children 12–23 months) and crepitations on auscultation, as assessed by the trained medical doctor.

### 2.7 Safety evaluation

Adverse events were solicited during home visits and at any visit to the study clinic within seven days following a vaccine dose. Solicited symptoms that included one major or two minor signs of illness were referred to the clinic for further evaluation. The frequency of clinic referrals and any clinic visit that occurred within seven days post-vaccination were compared by vaccine arm. Serious adverse events, defined as any clinical event that resulted in death, hospitalization, recovery with a disability, or was associated with convulsions, were assessed during the seven days post-vaccination and throughout follow-up.

### 2.8 Statistical analysis

The trial was designed to randomize 3410 children and was powered to detect a 10% VE against clinical pneumonia (to be presented elsewhere) and was based on the assumptions of 5% type I error, 20% type II error, and 15% participant attrition. This sample size was determined to be sufficient for >80% statistical power to detect a VE against laboratory-confirmed influenza of 34%, assuming a 10% attack rate.

The incidence of laboratory-confirmed influenza was compared between the trial arms. Episodes of influenza that occurred within 14 days of the first dose of vaccine were excluded. Child-time-at-risk was estimated starting 14 days after the first vaccine dose and continuing for one year after the start of the vaccination round, or once a child was re-vaccinated for a second season, moved out of the study area, withdrew consent, or died. Time spent ill was subtracted from child-time-at-risk.

Unadjusted Poisson regression was used to compare incidence rates of laboratory-confirmed influenza by vaccine arm. The vaccine efficacy (VE) was estimated as 100% × (1 − RR), where RR is the rate ratio estimated from Poisson regression. The regression model did not account for clustering of illness within children, since very few children had multiple episodes of laboratory-confirmed influenza in a given season. Fisher’s exact test was used to compare proportions of children with adverse events by trial arm.

Primary analyses were pre-specified and children were analyzed according to the vaccine they received. Vaccine efficacy was estimated for (1) all seasons, (2) among children in their first season of enrollment (first-time vaccinees), and (3) among children in their second season of enrollment (repeat vaccinees). Subgroup analyses were pre-specified to assess differences in VE against laboratory-confirmed influenza by age group, sex, and weight-for-age z-scores; no adjustments were made to type I error.

Secondary analyses were also pre-specified and included: (1) VE against infections with viruses antigenically matched to vaccine components, using HAI for antigenic characterization; (2) VE among a per protocol cohort, which included episodes and time-at-risk until a protocol deviation occurred; and (3) among all fully vaccinated children in the per protocol cohort. Protocol deviations included receiving a second dose of vaccine <29 days after a first dose, receiving a second dose >37 days after a first dose, not receiving a second dose during the first season of enrollment, receiving >1 dose during subsequent seasons of enrollment, switching trial arms during subsequent seasons of enrollment, and enrollment despite meeting exclusion criteria. The per protocol fully vaccinated cohort included episodes of laboratory-confirmed influenza that occurred ≥14 days after the second dose during a first season of enrollment or after the first dose during a subsequent season of enrollment.

Analyses were performed with SAS® version 9.3 (SAS Institute, Cary, NC) and R software (version 3.1.1). P-values <.05 were considered statistically significant.

### 2.9 Role of the funding source

Funding was provided by the Bill and Melinda Gates Foundation; however, the funder was not involved in protocol development or study conduct, analysis, write-up, or the decision on when and where to publish the findings.

## 3 Results

**Fig. 1:**
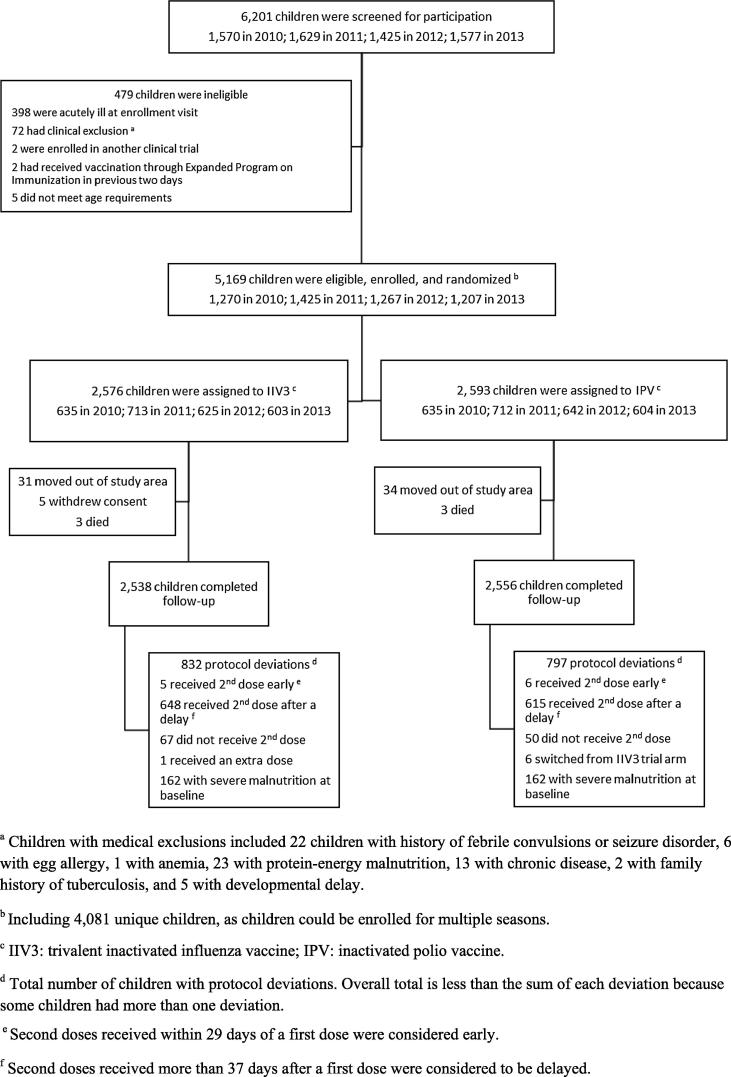
Consolidated Standards of Reporting Trials (CONSORT) diagram for recruitment, eligibility, enrollment, randomization, and follow-up of young children participating in a trial of trivalent inactivated influenza vaccine (IIV3) in Dhaka, Bangladesh, 2010–2014.

**Fig. 2:**
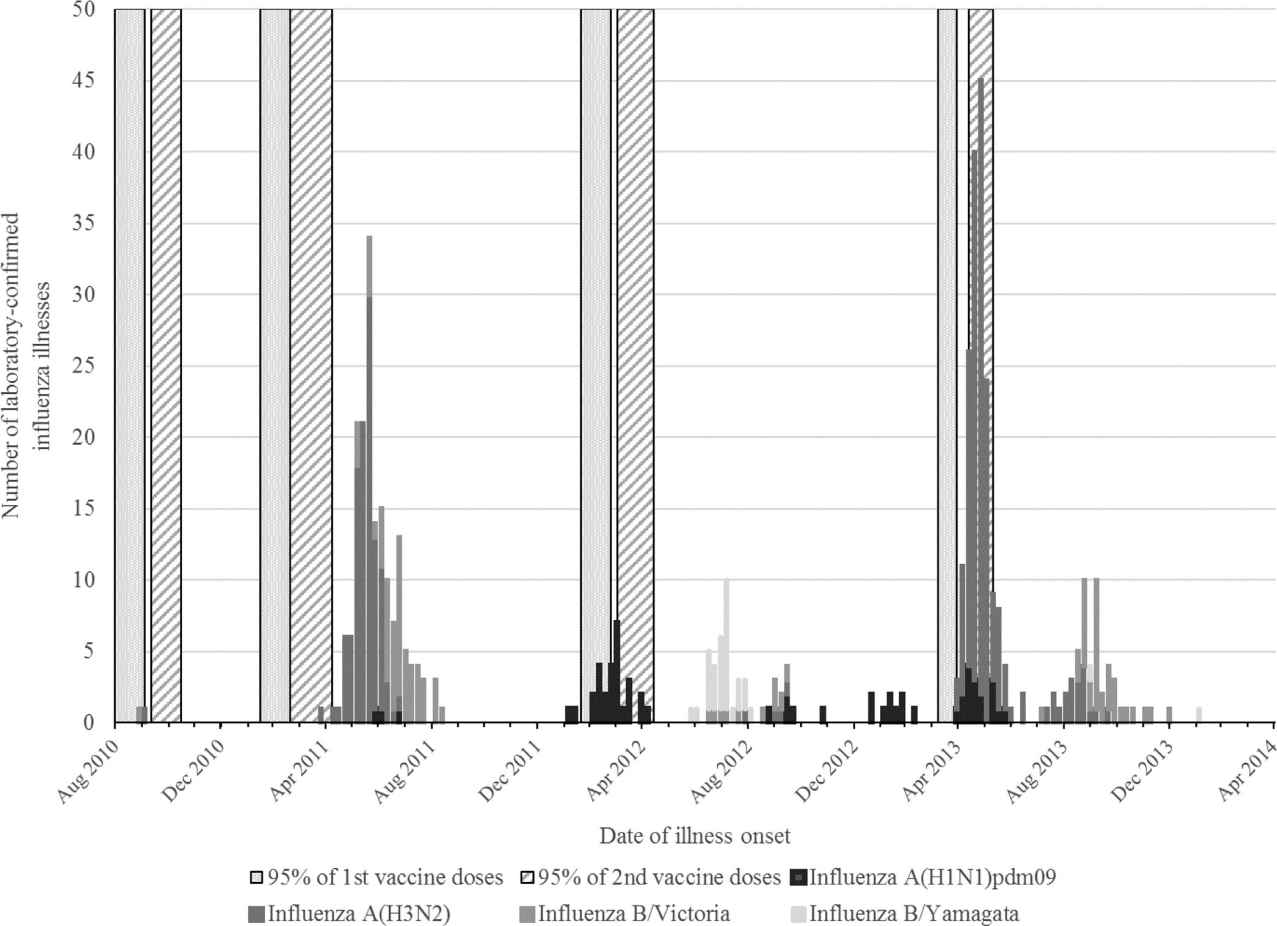
Periods of vaccination and laboratory-confirmed influenza virus infections among participants in a trial of trivalent inactivated influenza vaccine (IIV3) in children in Dhaka, Bangladesh, 2010–2014.

A total of 4081 children were enrolled and contributed 5,169 child-years of observation (Fig. 1 and Supplemental Fig. 1). Selected baseline characteristics were similar between the vaccine arms (Table 1). Among enrolled children, peaks of influenza A activity occurred between March and August and for influenza B between July and November each year (Fig. 2). From ongoing surveillance of influenza in the community, we observed that the majority of influenza A viruses that circulated during the study period were antigenically similar to the viruses in IIV3 (Supplemental Table 3). However, community surveillance indicated that the lineage of influenza B viruses that circulated in 2012, B/Yamagata, was not covered by the viral antigens in IIV3. In 2013, the vaccine composition changed to include an influenza B/Yamagata lineage virus. During 2013, however, influenza B/Victoria lineage viruses were seen in community surveillance indicating another season with mismatch of the B lineage antigen in the vaccine formulation.

**Table 1:** Baseline characteristics of children participating in a trial of trivalent inactivated influenza vaccine (IIV3) in Dhaka, Bangladesh, 2010–2014.

	IIV3 N = 2,576	IPV N = 2,593
Year, n (column %)		
2010	635 (25)	635 (24)
2011	713 (28)	712 (27)
2012	625 (24)	642 (25)
2013	603 (23)	604 (23)

Age, n (column %)		
6–11 months	767 (30)	786 (30)
12–17 months	870 (34)	847 (33)
18–23 months	939 (36)	960 (37)
Males, n (column %)	1,273 (49)	1,280 (49)

Weight-for-age z-scores		
Median z-score [IQR]	−1.4 [−2.1, −0.6]	−1.4 [−2.1, −0.7]
Normal (z−scores > −2), n (column %)	1,867 (72)	1,861 (72)
Moderate undernutrition (−3 < z-scores < −2)	547 (21)	570 (22)
Severe undernutrition (z-scores < −3)	162 (6)	162 (6)

Non-influenza childhood vaccines, n (column %)^a^		
Fully vaccinated	63 (2)	71 (3)
Partially vaccinated	2,484 (97)	2,498 (96)
Not vaccinated	22 (1)	21 (1)

IIV3: Trivalent Inactivated Influenza Vaccine; IPV: Inactivated Polio Vaccine; IQR: interquartile range.

a Children were considered fully vaccinated if they had received 1 dose of BCG vaccine, 3 doses of Diphtheria/Pertussis/Tetanus or 3 doses of pentavalent vaccine, 4 doses of oral polio vaccine, and, if at least 9 months old, 1 dose of measles vaccine. Children were considered partially vaccinated if they received any doses of any of the vaccines considered. Children were considered not vaccinated if they were not vaccinated with any of the vaccines considered. Ten children had insufficient information to determine vaccination status for the vaccines considered.

All children received at least one dose of their assigned vaccine and 68% of children (n = 3,540) completed the trial without deviation from protocol. Common deviations were receipt of a second dose more than 37 days after a first dose (1,262 children, 77% of all protocol deviations), severe malnutrition at baseline (231 children, 14%), no receipt of a second dose during a first enrollment season (117 children, 7%), receipt of a second dose less than 29 days after a first dose (11 children, <1%), vaccine received did not match randomization assignment (6 children, <1%), and receipt of a second dose during a subsequent enrollment season (2 children, <1%).

Overall, 4,067 episodes of illness occurred and a nasopharyngeal wash specimen was collected for 98% of episodes. Four hundred ninety-four episodes of illness (12.1% of 4067 illness episodes) were positive for influenza during the study period and 87 (17.6%) of the influenza-positive illnesses were diagnosed as clinical pneumonia. Among the 494 influenza virus infections, 430 viruses (87%) were isolated from specimens by viral culture and antigenically characterized. All influenza A(H1N1)pdm09 and A(H3N2) viruses isolated in the trial participants were antigenically similar to the vaccine antigens. All influenza B viruses were also antigenically similar to the vaccine antigens of the same lineage; however, 63 influenza B virus infections (44% of 143 influenza B virus infections detected) were considered mismatched to the vaccine because the opposite lineage was included in the trivalent formulation; 29 influenza B/Yamagata virus infections occurred when influenza B/Victoria virus antigens were included in the vaccine and 34 influenza B/Victoria virus infections occurred with influenza B/Yamagata virus antigens were included in the vaccine.

In the primary analysis, across all seasons, the incidence of laboratory-confirmed influenza was 10.0 episodes/100 child-years in the IIV3 arm and 14.5 episodes/100 child-years in the IPV arm resulting in a VE estimate of 31.1% (95% confidence interval (CI) 17.5%, 42.4%). There was significant VE of IIV3 against laboratory-confirmed influenza during the 2011 and 2013 seasons (Table 2). The incidence of influenza was lower in the IIV3 arm during the 2012 season, however this was not a statistically significant declined compared with the IPV arm. Study enrollment occurred at the end of influenza circulation for the 2010 season and influenza circulation had begun just prior to enrollment during the 2012 season. The VE was of similar magnitude against influenza A(H1N1)pdm09, influenza A(H3N2), and influenza B/Victoria virus infections, but only statistically significant against A(H3N2) and B/Victoria virus infections. The VE against influenza B/Yamagata lineage virus infections was lower compared with the other influenza virus infections and was not statistically significant. The VE against infections with viruses that were antigenically matched to the vaccine was 38.9% (95% CI: 24.6%, 50.6%). The VE against influenza virus infections antigenically matched to the vaccine was also higher compared with the VE against all influenza in each season (Table 2). The VE estimates stratified by virus subtype, lineage, and season are presented in the supplemental materials (Supplemental Table 4).

**Table 2:** Incidence of and vaccine efficacy against laboratory-confirmed influenza among children participating in a trial of trivalent inactivated influenza vaccine (IIV3) in Dhaka, Bangladesh, 2010–2014.

	IIV3			IPV			Vaccine efficacy (95% CI)^a^	P-value^b^
	N events	Child-years at risk	Incidence (per 100 child-years)	N events	Child-years at risk	Incidence (per 100 child-years)		
All influenza^c^	200	2,009	10.0	294	2,034	14.5	31.1 (17.5, 42.4)	<.001
2010 Season^d^	1	219	0.5	1	219	0.5	−0.2 (−1501, 93.7)	>.99
2011 Season	62	646	9.6	112	651	17.2	44.2 (24.0, 59.1)	<.001
2012 Season	41	599	6.8	49	621	7.9	13.3 (−31.3, 42.7)	.5
2013 Season	96	545	17.6	132	544	24.3	27.4 (5.6, 44.2)	.017

*Influenza virus subtype/lineage*								
Influenza A (H1N1pdm09)	25	2,011	1.2	38	2,039	1.9	33.3 (−10.5, 59.7)	.116
Influenza A (H3N2)	118	2,010	5.9	173	2,036	8.5	30.9 (12.7, 45.3)	.002
Influenza B/Victoria	40	2,011	2.0	61	2,038	3.0	33.5 (1.0, 55.4)	.045
Influenza B/Yamagata	13	2,012	0.6	18	2,039	0.9	26.8 (−49.4, 64.1)	.391

*Vaccine-matched influenza virus infections*^e^								
Overall	138	2,010	6.9	229	2,037	11.2	38.9 (24.6, 50.6)	<.001
2010 Season^d^	1	219	0.5	1	219	0.5	−0.2 (−1501, 93.7)	>.99
2011 Season	51	646	7.9	98	652	15.0	47.5 (26.4, 62.6)	<.001
2012 Season	11	599	1.8	23	621	3.7	50.4 (−1.7, 75.8)	.055
2013 Season	75	546	13.7	107	545	19.6	30.0 (6.0, 47.9)	.018

IIV3 = Trivalent Inactivated Influenza Vaccine; IPV = Inactivated Polio Vaccine.

a P-values compare IIV3 to IPV using unadjusted Poisson regression.

b Vaccine efficacy estimated as 100% × (1 − rate ratio).

c Influenza virus found in nasopharyngeal wash using RT-PCR.

d The 2010 season occurred from September 2010 to March 2011, 2011 season occurred from April 2011 to March 2012, 2012 season occurred from April 2012 to March 2013, and 2013 season occurred from April 2013 to March 2014.

e Influenza viruses from trial participants were antigenically characterized and compared with trivalent inactivated influenza vaccine antigen using hemagglutinin inhibition (HAI) assay. Viral isolates were considered similar to the vaccine (i.e. vaccine-matched) if antisera raised against the vaccine virus antigen reacted with the isolate with an HAI titer that was at least 4-fold higher than the reaction to other reference antisera.

**Fig. 3:**
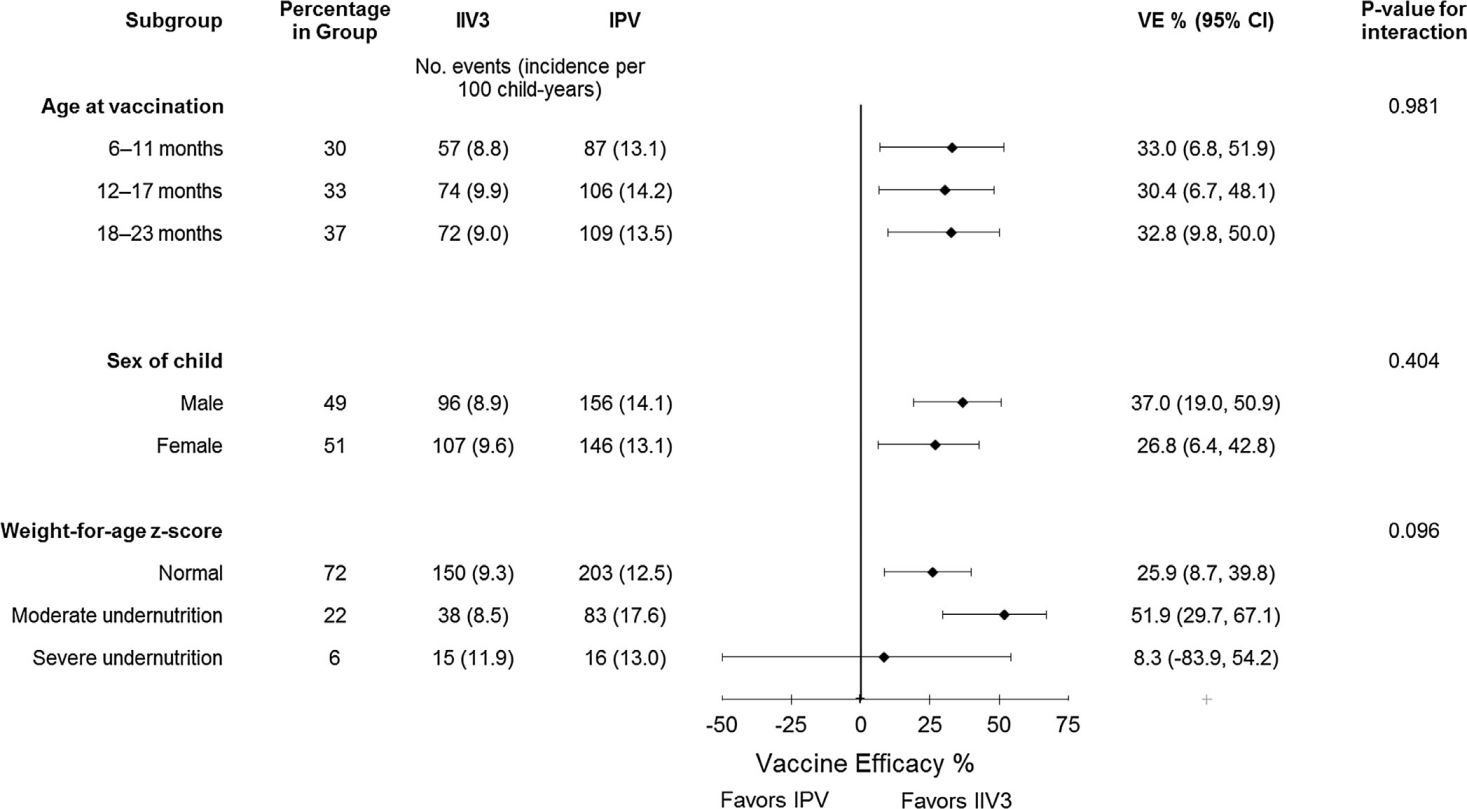
Vaccine efficacy estimates of trivalent inactivated influenza vaccine (IIV3) against laboratory-confirmed influenza by subgroups of children participating in a trial of IIV3 in Dhaka, Bangladesh, 2010–2014.

In subgroup analysis, the VE against laboratory-confirmed influenza was similar across 6-month age groups and by sex (Fig. 3). There were slight differences in VE estimates by weight-for-age categories of undernutrition, although this subgroup analysis did not reach statistical significance.

**Fig. 4:**
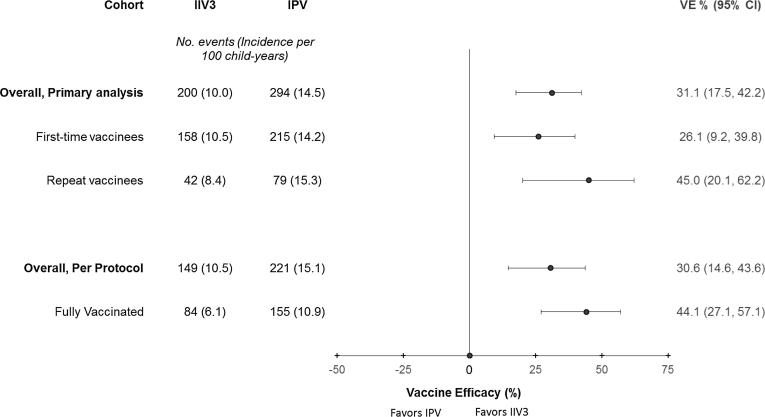
Vaccine efficacy estimates of trivalent inactivated influenza vaccine (IIV3) against laboratory-confirmed influenza by analysis cohort (primary or per protocol) of children participating in a trial of IIV3 in Dhaka, Bangladesh, 2010–2014.

The VE against influenza virus infection was similar in the first-time vaccinees compared to the primary analysis with all children, but was slightly higher among repeat vaccinees during a second season of enrollment (Fig. 4). The overall VE across seasons was also higher among children in the fully vaccinated, per protocol cohort than in the primary analysis. This finding was largely driven by differences in VE among the fully vaccinated in 2012. The VE against influenza in the overall primary analysis was non-significant in 2012, but was statistically significant in 2012 among fully vaccinated children without deviation from the protocol (VE = 58.6%, 95% CI: 10.9%, 80.7%; Supplemental Table 5). Other seasons had similar VE estimates in per protocol analysis compared with the primary analysis.

The difference in VE between the primary and fully vaccinated, per protocol analysis, in all seasons but particularly in 2012, follows from observed differences in the frequency of influenza among partially vaccinated children. In 2012, 34% of influenza episodes in the IIV3 arm and 10% of episodes in the IPV arm occurred between vaccine doses when children were only partially vaccinated. The frequency of influenza among the partially vaccinated was 11% in the IIV3 arm versus 0% in the IPV arm in the 2011 season and 32% in the IIV3 arm versus 17% in the IPV arm during the 2013 season. By virus type/subtype, 64% of influenza A(H1N1)pdm09, 3% of A(H3N2), and 2% of B virus infections, among children in the IIV3 arm, occurred during a time when the child was only partially vaccinated. Additionally, all of the episodes of influenza among partially vaccinated children in the IIV3 arm in 2012 occurred during the period of influenza A(H1N1)pdm09 circulation, which also supports why the VE against A(H1N1)pdm09 virus infection was non-significant in the primary analysis but was higher and statistically significant in the fully vaccinated, per protocol analysis (Supplemental Fig. 2).

Among children receiving IIV3, 6.2% experienced a clinical event within seven days of a vaccine dose with one major or two minor symptoms that prompted referral to the community clinic, which was similar to 6.7% among children receiving IPV (p = .463, Table 3). Serious adverse events, including death, hospitalization, disability, or an event associated with convulsions, within seven days of a vaccine dose occurred in 0.8% of children in each trial arm and their occurrence was not associated with vaccine type (p = 1.000). Throughout all of follow-up, less than 5% of children receiving IIV3 and IPV experienced a serious adverse event, the majority of which (92%) were classified as such because the event resulted in hospitalization. During the trial period, three deaths occurred in the IIV3 arm and three deaths occurred in the IPV arm. Of note, one death, in the IIV3 arm, occurred within seven days of a vaccine dose but was deemed as probably not related to the vaccine by the medical staff as it was associated with diarrheal illness.

**Table 3:** Adverse events among children participating in a trial of trivalent inactivated influenza vaccine (IIV3) in Dhaka, Bangladesh, 2010–2014.

	IIV3 (N = 2,576)	IPV (N = 2,593)	P-value^a^
	n events (%)	n events (%)	
Any adverse event^b^	160 (6.2)	175 (6.7)	.46

Any early serious adverse event^c^	21 (0.8)	22 (0.8)	1.00
Death	1 (0.04)	0 (0.0)	.50
Hospitalization	20 (0.8)	22 (0.8)	.88
Resulted in disability	0 (0.0)	0 (0.0)	1.00
Associated with convulsions	1 (0.0)	2 (0.1)	1.00

Any serious adverse event^d^	116 (4.5)	120 (4.6)	.84
Death	3 (0.1)	3 (0.1)	1.00
Hospitalization	110 (4.3)	108 (4.2)	.89
Resulted in disability	0 (0.0)	1 (0.04)	1.00
Associated with convulsions	5 (0.2)	9 (0.3)	.42

IIV3: Trivalent Inactivated Influenza Vaccine; IPV: Inactivated Polio Vaccine.

a P-value compares IIV3 to IPV using Fisher’s exact test.

b Any clinical event that occurred within 7 days of the first or second vaccine dose.

c Any serious adverse event that occurred within 7 days of a first or second vaccine dose.

d Any serious adverse event that occurred at any time during follow-up.

## 4 Discussion

Children under 2 years of age are particularly vulnerable to influenza-associated complications and are considered a risk group that should be targeted for influenza vaccination throughout the world [3]. We found that, among young children aged 6–23 months in urban Bangladesh during 2010–2014, vaccination with trivalent inactivated influenza vaccine (IIV3) was safe, significantly reduced incident episodes of laboratory-confirmed influenza by 31%, and significantly reduced incident infections with vaccine-matched viruses by 39%.

While statistically significant overall, the VE against laboratory-confirmed influenza varied by influenza season. During the 2010 season, vaccination occurred near the end of the typical season due to delays in vaccine shipment. We chose to continue with enrollment and vaccination that season despite the delay because influenza circulation can be prolonged in Dhaka; however, very few children in the trial were infected with influenza after the vaccination round began and we were unable to estimate an informative VE for this season. The highest observed VE was during the 2011 season, which was predominated by influenza A(H3N2) infections. In 2012, again due to delays in vaccine shipment, vaccination occurred during the midst of influenza A(H1N1)pdm09 circulation. Fewer cases were observed in each vaccine arm and more children were partially vaccinated at the time of infection, which likely contributed to the non-significant VE for the season in the primary analysis and against A(H1N1)pdm09 infections overall. Additionally, there was co-circulation of B lineage viruses in 2012 and a mismatch compared with the B lineage viruses in the vaccine, which likely further contributed to lower efficacy in that season. In 2012, the VE was much higher (50%) against viruses with matching antigens in the vaccine formulation, but was not statistically significant. In 2013, vaccination was completed prior to the first occurrences of influenza in the study population; but, again, there was a mismatch between the lineage of circulating influenza B viruses and vaccine virus. Thus, our vaccine efficacy measurements are likely under-estimates of optimal roll out of well-matched vaccine.

The point estimates for VE did not vary much by type or subtype of influenza infection; however, the VE was statistically significant against influenza A(H3N2) and influenza B/Victoria virus infections but was not significant against influenza A(H1N1)pdm09 or B/Yamagata virus infections in the primary analysis. The study period was comprised of mostly influenza A(H3N2) and B/Victoria virus infections, thus we were likely underpowered to detect a significant VE against influenza A(H1N1)pdm09. Additionally, when we stratified infections by whether the child was fully or partially vaccinated at the time of infection, we saw many more A(H1N1)pmd09 infections among children who were partially vaccinated than for the other types and subtypes of infection.

Putting our overall findings in context, one other trial of IIV3 among children aged 6–23 months reported significant VE against any culture-confirmed influenza during the 1999–2000 influenza season in the US (66% VE, 95% CI: 34, 82%) [4]. However, non-significant VE was reported during the subsequent season [4] and from two additional trials with all or the majority of participants in this age group [5], [6]. Several observational studies have reported on vaccine effectiveness in this young age group during non-pandemic seasons from 2003 to 2011, all within high-income countries [15], [16], [17], [18], [19], [20], [21], [22]. Of the nine included seasons, significant VE estimates, ranging from 25% to 86%, were reported in five seasons [15], [16], [17], [19], [20]. The variable effect of IIV3 may be related to how timing of vaccine administration matches with the peak and duration of transmission in any given season, and highlights the need to adequately plan and design influenza vaccine studies. The trial in Dhaka was designed to include three seasons; however, because of the delayed start of vaccination in 2010, enrollment was extended in 2011 to a fourth season, after blinded interim review. Without this additional season, our estimate of VE would have been limited to one season in which vaccination was completed prior to influenza circulation in the area.

Compared with the VE among adults or in older children, the modest VE we observed may be reflective of lower immunogenicity of non-adjuvanted inactivated influenza vaccines in young children [5], [23], [24], [25], [26], [27]. Phase II immunogenicity studies have shown that young children 6–11 months have lower geometric mean antibody titers and lower rates of seroprotection than older children [5], [23], [25], [28], [29]. Older children may also have higher VE than young children because of a boosting effect of repeated vaccination or natural infections. We did see slightly higher VE against laboratory-confirmed influenza during a child’s second season of enrollment, although the confidence intervals for repeated vaccination overlapped with those for the overall cohort and the cohort of first-time vaccinees. A similar pattern was reported in an observational study of children aged 2–8 years in the United States [30] suggesting that young children may get an additional boost from second-year vaccination.

We also observed a slightly higher VE for fully vaccinated children compared with the overall cohort. Through further investigation, the increase in VE was largely driven by increased VE among the fully vaccinated during the 2012 season when vaccination occurred during circulation of A(H1N1)pdm09. The study was designed to end vaccination prior to the start of influenza circulation in the area, which had historically been between April and October; and May has been suggested as the optimal time of vaccination based on data from 2006 to 2011 [11]. However, influenza circulation in the tropics can be sporadic and there was early circulation of A(H1N1)pdm09 in Dhaka in 2012. Many children were exposed to influenza A(H1N1)pdm09 prior to being fully vaccinated with two doses of IIV3. This context could have led to higher VE among the fully vaccinated because there is greater protection against influenza conferred by two doses of vaccine given during a first season, rather than one, or because the observation time for the fully vaccinated children started later and therefore included fewer infections than the primary analytic cohort did. During the 2011 and 2013 seasons, the VE among the fully vaccinated was similar to that of the primary analytic cohort, perhaps because both vaccine doses were administered before influenza was widely circulating, thus making those two cohorts virtually identical.

In addition to the limitations of this trial that have been presented above, a high proportion of children had protocol deviations and did not receive a second, booster dose within one month of their initial dose. The results of the per protocol analysis were similar to those of the primary analysis, so these deviations are unlikely to bias our conclusions. However, they do underscore the difficulty of 2-dose vaccination strategies for influenza, with often-sporadic circulation in tropical and subtropical settings, and encourage efforts to find optimal strategies for vaccine administration, including timing of vaccination, and more immunogenic and universal vaccines for young children.

In summary, we found that IIV3 significantly reduced the incidence of influenza in young children in Bangladesh. These findings support the recommendation for yearly vaccination of young children against influenza and confirm that these children likely benefit from an additional booster dose of inactivated vaccine in their first year of vaccination. As very young children are particularly vulnerable to severe influenza complications, alternative influenza vaccines with greater immunogenicity and efficacy should be evaluated, including vaccines with adjuvants or standard adult doses. While alternative influenza vaccines are being explored, inactivated influenza vaccines remain the only licensed vaccine for children aged 6–23 months and are currently the best tool to prevent infection among very young children who are at risk for severe complications from influenza.

## Contributors

All authors meet the criteria for authorship according to the International Committee of Medical Journal Editors (ICJME). WAB, AMF, MS, SL, and JB conceptualized the trial objectives and design; WAB wrote the trial protocol; DG was the primary study coordinator, overseeing training, conduct, and data management; ATS, SY, NP, and KN were clinical staff responsible for clinical care and management of trial participants; MR, DA, MZR, and MB, were responsible for laboratory activities, including diagnostic testing and influenza virologic surveillance during the trial; MAR and LHM developed the analysis plan with input from WAB; MAR did the statistical analysis with input from LHM; MAR wrote the first draft of the manuscript with input from WAB and AMF; all authors reviewed subsequent drafts of the manuscript and approved of the draft submitted for publication.

## Conflict of interest

None.

## Funding

This work was supported by a grant from the Bill & Melinda Gates Foundation, Seattle, WA, United States [Grant No. GR-00787].
