# Supplementary material for: Efficacy of trivalent influenza vaccine against laboratory-confirmed influenza among young children in a randomized trial in Bangladesh
Source: Vaccine. 2017 Dec 15;35(50):6967–76. doi: 10.1016/j.vaccine.2017.10.074 (PMC5723570; doi:10.1016/j.vaccine.2017.10.074)
Supplement: Supplementary data 2 [file mmc2.docx]

**Supplemental Figure 1:** Recruitment, screening, enrollment, randomization, and follow-up, by season, for young children participating in a trial of trivalent inactivated influenza vaccine (IIV3) in Dhaka, Bangladesh, 2010–2014.

**
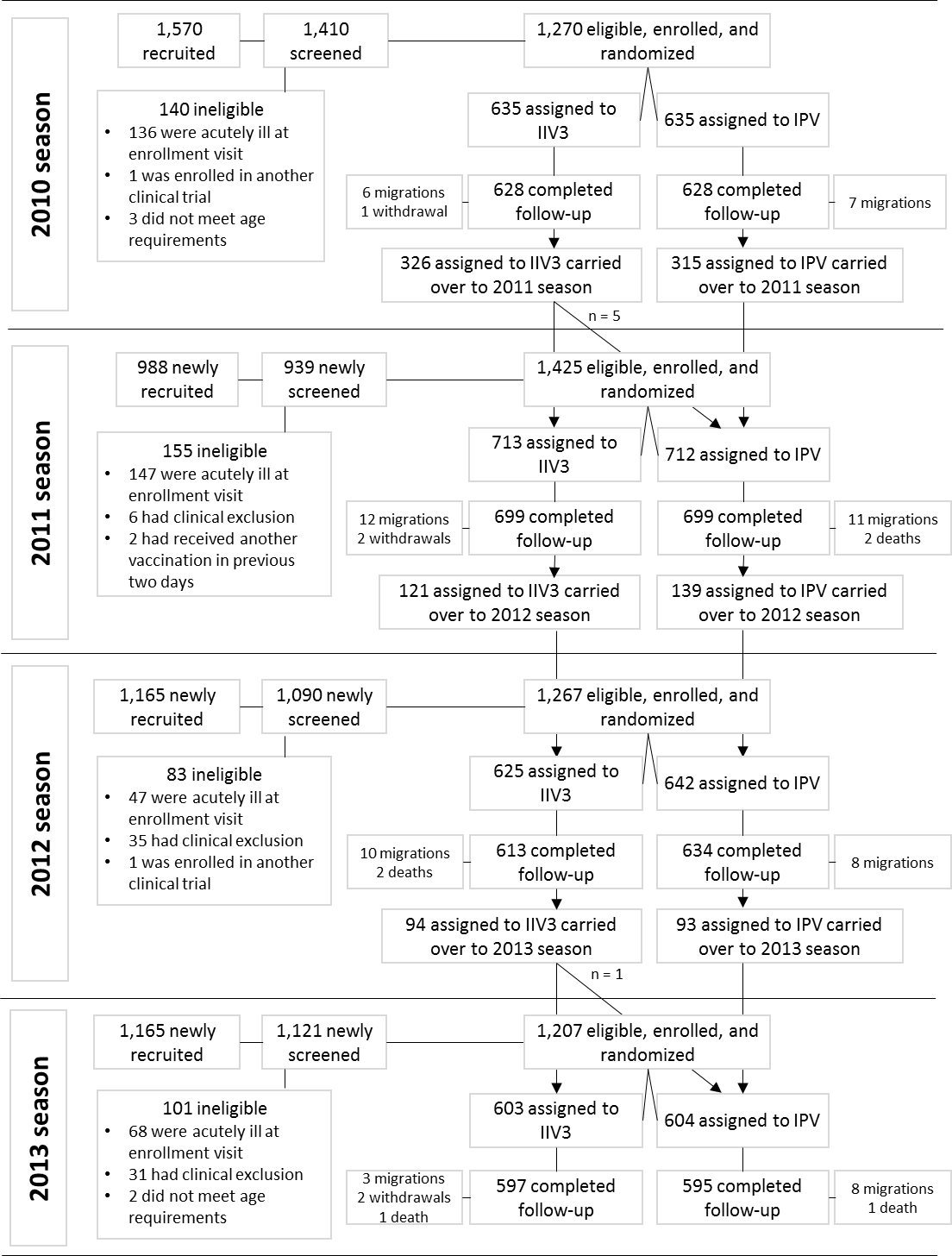
**

**Supplemental Figure 2:** Influenza episodes by season and vaccination status of young children participating in a trial of trivalent inactivated influenza vaccine (IIV3) in Dhaka, Bangladesh, 2010–2014.


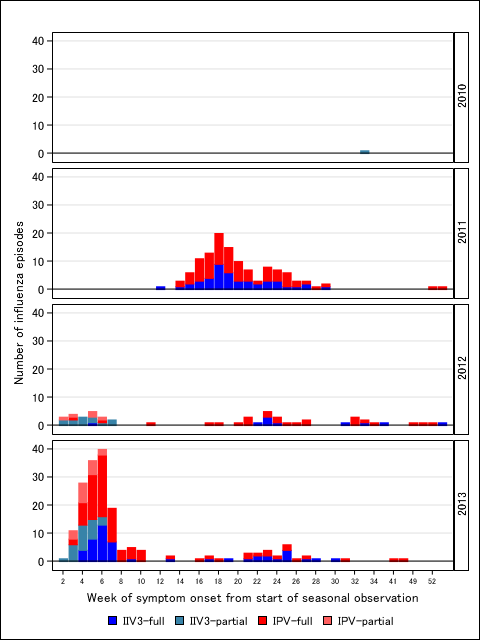


**Supplemental Table 1:** Vaccine lot numbers used in a randomized trial of trivalent influenza vaccine in children in the Kamalapur area of Dhaka, Bangladesh, 2010–2014

| Year | Vaccine | Lot Number | Expiry Date |
| --- | --- | --- | --- |
| 2010 | VAXIGRIP | G7051 | 28.02.2011 |
| 2010 | IMOVAX | D0238 | 28.02.2011 |
| 2011 | VAXIGRIP | G7111 | 30.06.2011 |
| 2011 | IMOVAX | D6082 | 30.09.2011 |
| 2012 | VAXIGRIP | H7093 | 31.05.2012 |
| 2012 | IMOVAX | E1276, G0445 & G7200 | 30.11.2012, 31.08.2013 & 30.11.2013 |
| 2013 | VAXIGRIP | J7331 | 31.10.2013 |
| 2013 | IMOVAX | H7080 | 30.04.2013 |

VAXIGRIP was the trade name for trivalent inactivated influenza vaccine from Sanofi-Pasteur.

IMOVAX was the trade name for the inactivated polio vaccine from Sanofi-Pasteur.

**Supplemental Table 2:** Clinical case definitions used in a randomized trial of trivalent inactivated influenza vaccine in children aged <2 years in Dhaka, Bangladesh, 2010–2014.

| **Diagnosis** | **Clinical case definition** |
| --- | --- |
| Pneumonia | 2 criteria:  1) Age-specific tachypnoea (≥60/min if < 2 months; ≥50/min if 2 – 11 months; ≥40/min if 12 – 59 months);  2) Crepitations on auscultation.  Severe pneumonia:  Pneumonia, above, plus chest indrawing  Very severe pneumonia:  Pneumonia, above, plus at least one danger sign (central cyanosis, severe respiratory distress, convulsions, altered mental status, head nodding, nasal flaring, grunting, inability to drink, lethargy, or vomiting) |
| Meningitis | Fever (axillary temperature >37.5 C or rectal >38 C), and presence of neck stiffness or altered consciousness or other meningeal signs or petechial purpural rash or fever accompanied by bulging fontanelle |
| Sepsis | Fever (axillary temperature >37.5 C or rectal >38 C) and presence of any of general danger sign/s (including inability to drink or breast feed, persistent vomiting, convulsions, lethargy, and altered  consciousness). |
| Upper respiratory infection | Fever (axillary temperature >37.5 C or rectal >38 C), cough and rhinorrhoea |
| Otitis media | Injection (rubour) of the tympanic membrane and/or reduced movement on insufflation  Suppurative otitis media:  Purulent (if acute) or clear (if chronic) fluid from the ear |
| Sinusitis | Purulent nasal discharge or nasal discharge persisting more than 14 days with or without cough and fever |
| Typhoid | History or documented fever (38.0C or more) for >=3 consecutive days, coated tongue, abdominal pain, diarrhea or constipation etc. |
| Diarrhea | Loose/Watery stool ≥ 3 times /24 hour |
| Bronchiolitis | Wheezing on auscultation, first attack of wheezing, documented fever or history of fever, age specific elevated respiratory rate |
| Catarrhal signs | Fever any duration with any two of cough, runny nose, or sore throat |

**Supplemental Table 3:** Antigenic characterization of influenza viruses detected through community disease surveillance in the Kamalapur area of Dhaka, Bangladesh, 2010–2014.

|  | **Total, n (%)** | **n (%) antigenically similar to the vaccine virus** | **n (%) antigenically different from vaccine virus** |
| --- | --- | --- | --- |
| **2010 season** |  |  |  |
| Number of influenza positive specimens | 26 |  |  |
| **Influenza A** | **24 (92)** |  |  |
| A(H1N1)pdm09 | 3 (13) | 3 (100) | 0 (0) |
| A(H3N2) | 21 (87) | 21 (100) | 0 (0) |
| **Influenza B** | **2 (8)** |  |  |
| B Victoria lineage | 1 (50) | 1 (100) | 0 (0) |
| B Yamagata lineage | 1 (50) | 0 (0) | 1 (100) |
| **2011 season** |  |  |  |
| Number of influenza positive specimens | 334 |  |  |
| **Influenza A** | **80 (24)** |  |  |
| A(H1N1)pdm09 | 20 (25) | 20 (100) | 0 (0) |
| A(H3N2) | 60 (75) | 60 (100) | 0 (0) |
| **Influenza B** | **254 (76)** |  |  |
| B Victoria lineage | 254 (100) | 254 (100) | 0 (0) |
| B Yamagata lineage | 0 (0) |  | -- |
| **2012 season** |  |  |  |
| Number of influenza positive specimens | 402 |  |  |
| **Influenza A** | **206 (51)** |  |  |
| A(H1N1)pdm09 | 189 (92) | 189 (100) | 0 (0) |
| A(H3N2) | 17 (8) | 16 (94) | 1 (6) |
| **Influenza B** | **196 (49)** |  |  |
| B Victoria lineage | 46 (23) | 46 (100) | 0 (0) |
| B Yamagata lineage | 150 (77) | 0 (0) | 150 (100) |
| **2013 season** |  |  |  |
| Number of influenza positive specimens | 447 |  |  |
| **Influenza A** | **320 (72)** |  |  |
| A(H1N1)pdm09 | 66 (21) | 66 (100) | 0 (0) |
| A(H3N2) | 254 (79) | 254 (100) | 0 (0) |
| **Influenza B** | **127 (28)** |  |  |
| B Victoria lineage | 120 (94) | 0 (0) | 120 (100) |
| B Yamagata lineage | 7 (6) | 7 (100) | 0 (0) |

**Supplemental Table 4:** Incidence of and vaccine efficacy of IIV3 against laboratory-confirmed influenza, by virus subtype and lineage, among all children participating in a trial in Dhaka, Bangladesh, 2010–2014.

|  | **IIV3** | | |  | **IPV** | | |  |  |
| --- | --- | --- | --- | --- | --- | --- | --- | --- | --- |
|  | N events | Child-years at risk | Incidence (per 100 child-years) |  | N events | Child-years at risk | Incidence (per 100 child-years) | Vaccine Efficacy (95% CI) ^a^ | P-value ^b^ |
| Influenza A(H1N1)pdm09 ^c^ | 25 | 2,011 | 1.2 |  | 38 | 2,039 | 1.9 | 33.3 [-10.5, 59.7] | 0.12 |
| 2010 Season ^d^ | 0 | 219 | 0.0 |  | 0 | 219 | 0.0 | -- | -- |
| 2011 Season | 0 | 647 | 0.0 |  | 6 | 653 | 0.9 | -- | -- |
| 2012 Season | 21 | 599 | 3.5 |  | 19 | 621 | 3.1 | -14.5 [-113, 38.4] | 0.67 |
| 2013 Season | 4 | 546 | 0.7 |  | 13 | 546 | 2.4 | 69.3 [5.8, 90.0] | 0.039 |
|  |  |  |  |  |  |  |  |  |  |
| Influenza A(H3N2) ^c^ | 118 | 2,010 | 5.9 |  | 173 | 2,036 | 8.5 | 30.9 [12.7, 45.3] | 0.002 |
| 2010 Season ^d^ | 1 | 219 | 0.5 |  | 0 | 219 | 0.0 | -- | -- |
| 2011 Season | 39 | 646 | 6.0 |  | 72 | 651 | 11.1 | 45.4 [19.4, 63.0] | 0.002 |
| 2012 Season | 2 | 600 | 0.3 |  | 2 | 621 | 0.3 | -3.6 [-636, 85.4] | 0.972 |
| 2013 Season | 76 | 546 | 13.9 |  | 99 | 545 | 18.2 | 23.4 [-3.3, 43.2] | 0.081 |
|  |  |  |  |  |  |  |  |  |  |
| Influenza B/Victoria ^c^ | 40 | 2,011 | 2.0 |  | 61 | 2,038 | 3.0 | 33.5 [1.0, 55.4] | 0.045 |
| 2010 Season ^d^ | 0 | 219 | 0.0 |  | 1 | 219 | 0.5 | -- | -- |
| 2011 Season | 22 | 647 | 3.4 |  | 34 | 652 | 5.2 | 34.7 [-11.6, 61.8] | 0.119 |
| 2012 Season | 2 | 600 | 0.3 |  | 8 | 621 | 1.3 | 74.1 [-22.0, 94.5] | 0.088 |
| 2013 Season | 16 | 546 | 2.9 |  | 18 | 546 | 3.3 | 11.2 [-74.1, 54.7] | 0.729 |
|  |  |  |  |  |  |  |  |  |  |
| Influenza B/Yamagata ^c^ | 13 | 2,012 | 0.6 |  | 18 | 2,039 | 0.9 | 26.8 [-49.4, 64.1] | 0.391 |
| 2010 Season ^d^ | 0 | 219 | 0.0 |  | 0 | 219 | 0.0 | -- | -- |
| 2011 Season | 0 | 647 | 0.0 |  | 0 | 653 | 0.0 | -- | -- |
| 2012 Season | 13 | 599 | 2.2 |  | 16 | 621 | 2.6 | 15.8 [-75.0, 59.5] | 0.645 |
| 2013 Season | 0 | 547 | 0.0 |  | 2 | 546 | 0.4 | -- | -- |

IIV3 = Trivalent Inactivated Influenza Vaccine; IPV = Inactivated Polio Vaccine.

^a^ Vaccine efficacy estimated as 100% x (1 - rate ratio). Vaccine efficacy was undefined when 0 events occurred in either the IIV3 or the IPV arm.

^b^ P-values compare IIV3 to IPV using unadjusted Poisson regression.

^c^ Influenza viruses were detected in nasopharyngeal wash specimens using RT-PCR. Influenza B virus lineage was determined from cell culture isolates using hemagglutinin inhibition assay, as described in the Methods.

^d^ The 2010 season from occurred from September 2010 to March 2011, 2011 season occurred from April 2011 to March 2012, 2012 season occurred from April 2012 to March 2013, and 2013 season occurred from April 2013 to March 2014.

**Supplemental Table 5:** Incidence of and vaccine efficacy of IIV3 against laboratory-confirmed influenza among 3,540 children without protocol deviation in a trial in Dhaka, Bangladesh, 2010–2014.

|  | IIV3 | | |  | IPV | | |  |  |
| --- | --- | --- | --- | --- | --- | --- | --- | --- | --- |
|  | N events | Child-years at risk | Incidence |  | N events | Child-years at risk | Incidence | Vaccine Efficacy | P-value ^b^ |
|  |  |  | (per 100 child-years) |  |  |  | (per 100 child-years) | (95% CI) ^a^ |  |
| **All Children** |  |  |  |  |  |  |  |  |  |
| All Influenza ^c^ | 149 | 1,419 | 10.5 |  | 221 | 1,460 | 15.1 | 30.6 [14.6, 43.6] | <0.001 |
| 2010 Season | 1 | 153 | 0.7 |  | 0 | 155 | 0.0 | -- | -- |
| 2011 Season | 47 | 474 | 9.9 |  | 80 | 477 | 16.8 | 40.9 [15.3, 58.8] | 0.004 |
| 2012 Season | 22 | 347 | 6.3 |  | 30 | 380 | 7.9 | 19.6 [-39.4, 53.6] | 0.438 |
| 2013 Season | 79 | 445 | 17.7 |  | 111 | 448 | 24.8 | 28.4 [4.5, 46.4] | 0.023 |
|  |  |  |  |  |  |  |  |  |  |
| A(H1N1)pdm09 | 19 | 1,420 | 1.3 |  | 25 | 1,463 | 1.7 | 21.7 [-42.1, 56.9] | 0.421 |
| A(H3N2) | 96 | 1,419 | 6.8 |  | 137 | 1,461 | 9.4 | 27.9 [6.4, 44.4] | 0.014 |
| B/Victoria | 31 | 1,420 | 2.2 |  | 46 | 1,463 | 3.1 | 30.6 [-9.4, 56.0] | 0.116 |
| B/Yamagata | 3 | 1,421 | 0.2 |  | 12 | 1,463 | 0.8 | 74.3 [8.8, 92.7] | 0.036 |
| **Fully-vaccinated Children** | |  |  |  |  |  |  |  |  |
| All Influenza ^c^ | 84 | 1,374 | 6.1 |  | 155 | 1,418 | 10.9 | 44.1 [27.1, 57.1] | <0.001 |
| 2010 Season | 0 | 141 | 0.0 |  | 0 | 145 | 0.0 | -- | -- |
| 2011 Season | 47 | 463 | 10.1 |  | 81 | 465 | 17.4 | 41.7 [16.4, 59.3] | 0.003 |
| 2012 Season | 9 | 330 | 2.7 |  | 24 | 364 | 6.6 | 58.6 [10.9, 80.7] | 0.024 |
| 2013 Season | 28 | 440 | 6.4 |  | 50 | 443 | 11.3 | 43.7 [10.5, 64.5] | 0.015 |
|  |  |  |  |  |  |  |  |  |  |
| A(H1N1)pdm09 | 2 | 1,375 | 0.1 |  | 16 | 1,420 | 1.1 | 87.1 [43.9, 97.0] | 0.006 |
| A(H3N2) | 48 | 1,374 | 3.5 |  | 79 | 1,419 | 5.6 | 37.3 [10.2, 56.2] | 0.011 |
| B/Victoria | 29 | 1,265 | 2.3 |  | 45 | 1,306 | 3.4 | 33.5 [-6.1, 58.3] | 0.087 |
| B/Yamagata | 3 | 1,265 | 0.2 |  | 12 | 1,307 | 0.9 | 74.2 [8.5, 92.7] | 0.036 |

IIV3 = Trivalent Inactivated Influenza Vaccine; IPV = Inactivated Polio Vaccine.

^a^ Vaccine efficacy estimated as 100% x (1 - rate ratio).

^b^ P-values compare IIV3 to IPV using unadjusted Poisson regression.

^c^ Influenza viruses were detected in nasopharyngeal wash specimens using RT-PCR.
